# The applicability of a structured learning programme focusing on improving observational competencies to strengthen patient safety: a qualitative study with professionals in primary healthcare services

**DOI:** 10.1186/s12913-025-12692-y

**Published:** 2025-04-15

**Authors:** Emilie Alfstad, Veronica Lockertsen, Anne-Kari Johannessen, Anne Werner

**Affiliations:** 1 Department of Nursing and Health Promotion, Faculty of Health Sciences, OsloMet– Oslo Metropolitan University, P.O. Box 4, St. Olavs plass, Oslo, Norway; 2 HØKH– Health Services Research Unit, Akershus University Hospital (Ahus), P.O. Box 1000, Lørenskog, N- 1478 Norway

**Keywords:** Primary healthcare, Healthcare professionals, Observational competence, Competence programme, Patient deteriorations, Patient safety, Qualitative study, Collaboration, Situated learning

## Abstract

**Background:**

Primary healthcare in many Western countries faces increased patient care needs due to shorter hospital stays and an ageing population suffering from complex conditions. A shortage of qualified professionals jeopardises the quality of care in primary healthcare settings. Literature indicates that the quality of care and the occurrence of adverse events are linked to the observational competencies of healthcare professionals. In Norway, patient safety competence programmes, such as ClinObsMunicipality, have been developed to improve healthcare professionals’ observational competencies in recognising and responding to clinical deterioration, thereby ensuring safety in primary healthcare. In this study, we aimed to explore and describe how healthcare professionals experienced and perceived learning and training in this competence-building programme. Specifically, we focused on their reflections on its applicability in clinical practice.

**Design:**

Aqualitative study was conducted. In preparing the manuscript, we applied the checklist guidelines for the Consolidated Criteria for Reporting Qualitative Research.

**Method:**

The study is based on 17 individual interviews with healthcare professionals from different primary healthcare settings. Data were analysed using Malteruds’ systematic text condensation, a thematic cross-case analysis.

**Results:**

We identified three categories illustrating how healthcare professionals experienced learning and training in the competence-building programme and its applicability for clinical practice: (1) Shared base of competence through practice-based group-learning with colleagues; (2) Enhanced clinical communication: The impact and applicability of standardised language; (3) From Colleagues to team: Increased autonomy and collaboration.

**Conclusion:**

This study highlights that healthcare professionals experienced learning and training in the competence programme as applicable to their clinical practice. Group-learning activities fostered a supportive environment where participants could engage with cases and scoring tools relevant to their clinical settings. This approach enhanced their observational competencies, deepened their understanding of team roles and strengthened interprofessional communication and collaboration, which may positively impact patient care and safety in primary healthcare settings. While the programme empowered healthcare professionals through increased autonomy, it also uncovered hesitance in critical situations among some participants, suggesting complex awareness and the urgency of timely interventions.

**Supplementary Information:**

The online version contains supplementary material available at 10.1186/s12913-025-12692-y.

## Background

Across many Western countries, primary healthcare services experience increased patient care needs, driven by shorter hospitalisations and an ageing population with chronic and more complex conditions [1–4]. Healthcare professionals, including registered nurses (RNs), assistant nurses (ANs) and social educators, play a fundamental role in detecting and responding to deteriorating frail older patients, where observational competence is crucial for providing appropriate patient care [5]. Therefore, primary healthcare must improve the observational competencies of healthcare professionals to meet quality standards and achieve better patient outcomes [4–6]. In recent decades, European health reforms have aimed to enhance patient safety and improve primary healthcare quality [7, 8]. These reforms often redistribute tasks and responsibilities from secondary to primary healthcare providers, emphasising the importance of healthcare professionals’ competencies when delivering effective patient care [9]. However, policy and research continue to highlight significant challenges related to the adequacy of existing competencies among primary healthcare professionals to meet the complex care needs of the ageing population [10–12]. This inadequacy raises essential questions related to the ability of primary healthcare professionals to ensure and maintain patient safety.

The World Health Organisation (WHO) defines patient safety as a variety of activities designed to cultivate a culture of safety, establish processes and procedures, and implement technologies and environments that collectively reduce the risk of injury to patients [13]. It has been shown that the quality of care, patient outcomes and the incidence of adverse events are associated with the competencies of healthcare professionals [14]. A study on systematic observations of frail older patients in home care settings found significant variations in how healthcare professionals measured patients’ vital signs and recognised deterioration [15]. Many displayed a lack of awareness in this area. Early recognition and timely responses to clinical deterioration by observing vital signs are crucial for enhancing patient safety [15]. Therefore, there is a need for ongoing competence development in this field. Registered Nurses (RNs) play a pivotal role in this regard, with clinical competencies that are crucial for maintaining patient safety. Research has demonstrated that professional education and experience positively impact RN competencies, further underscoring their importance in primary healthcare [16]. Nevertheless, many regions in Western countries face a shortage of health workers, particularly skilled professionals, which jeopardises the quality of care in primary healthcare settings [17]. This raises substantial concerns about the overall quality and safety of provided care. To adequately address these challenges, enhancing the observational competencies of primary healthcare professionals is essential. Moreover, healthcare professionals identify communication and competence as essential factors to reduce safety risks [18]. Ongoing training and collaborative learning approaches have emerged as effective strategies for improving patient safety and quality in primary healthcare settings [19, 20].

**Fig. 1:**
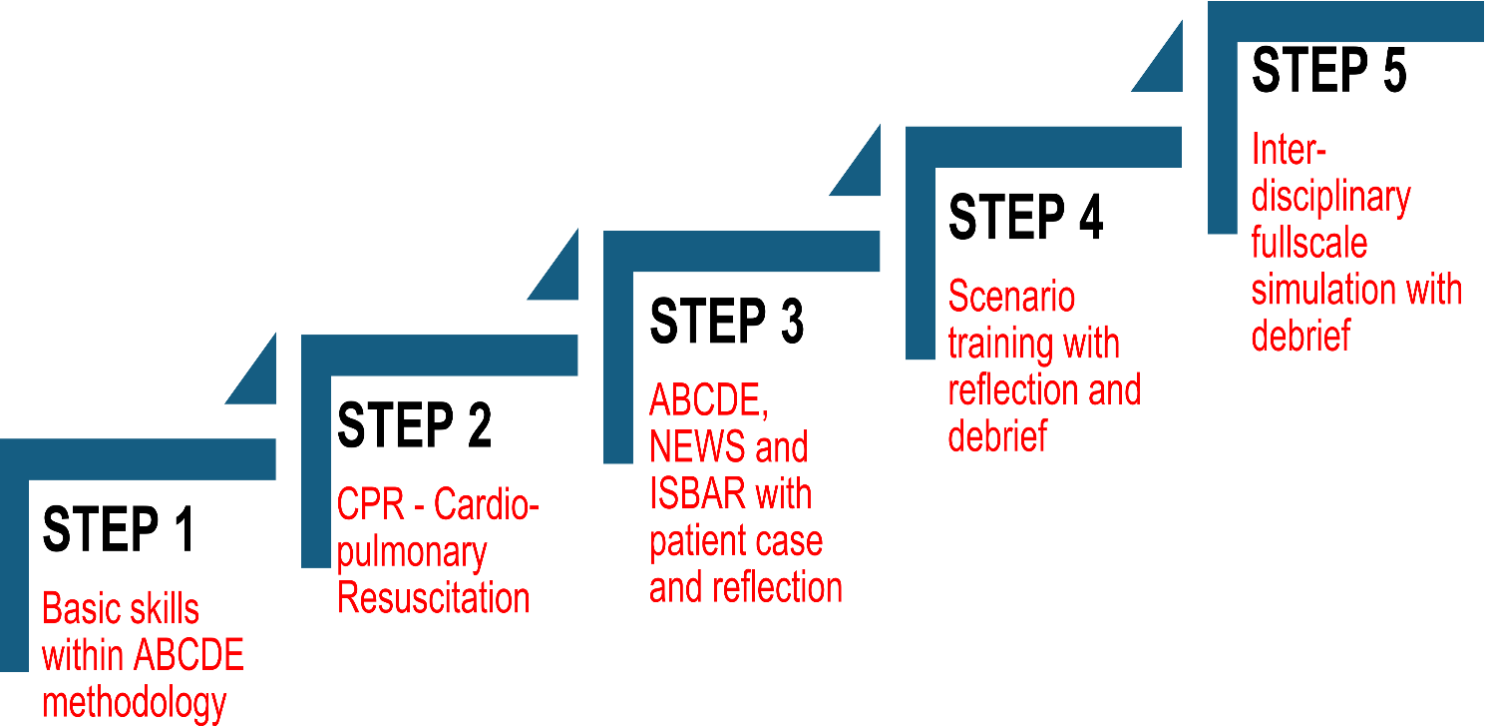
ClinObsMunicipality, a step-by-step competence model*

The ClinObsMunicipality competence-improvement programme was launched in 2020 as a part of the Norwegian Patient Safety Programme [21, 22]. This initiative is a proactive response by Norwegian municipalities to policy recommendations for early detection of patient deterioration, with the aim of enhancing patient safety in primary healthcare [22]. The programme is a step-by-step competency model that focuses on improving healthcare personnel’s observational and assessment competencies in primary healthcare settings. This involves evaluating a patient’s clinical condition to determine whether their physical or mental state has changed or deteriorated and deciding on necessary interventions [22, 23]. The programme follows the Train-The-Trainer model, where leaders select potential ‘instructors’ in the relevant field or workplaces [24]. These instructors receive training to teach colleagues, aiming to cultivate committed professionals who can lead local competence development in selected topics [25, 26]. ClinObsMunicipality provides theoretical and practical training and activities in observational competencies about the principles of ABCDE (i.e., Airways, Breathing, Circulation, Disability, and Exposure) and CPR (i.e., Cardiopulmonary Resuscitation) [21]. It also includes training in the communication tool ISBAR (i.e., Identification, Situation, Background, Assessment, and Recommendation) for effective and safe delivery of information in acute situations with deterioration of patients’ condition [22]. In addition, the competence programme includes training in a risk assessment tool known as the National Early Warning Score (NEWS), which assesses patients’ conditions in six dimensions: [27]. See Fig. 1.^1^

There is a notable gap in research focusing on patient safety within the primary healthcare context [12, 28]. Furthermore, knowledge is scarce regarding how changes in service delivery and the transition of care tasks impact healthcare professionals’ roles and practices in clinical work [29, 30]. There are also limited experiences of competence-improvement programmes and group-learning activities to enhance healthcare professionals’ observational competencies and increase patient safety in primary healthcare services. To address this gap, this study aimed to explore and describe how healthcare professionals experienced learning and training in the competence-building programme for primary healthcare services. Specifically, we focused on their reflections on the applicability in clinical practice.

### Theoretical perspectives

ClinObsMunicipality emphasises group-learning as a method for increasing observational competencies by combining theoretical knowledge with practical group-learning activities among primary healthcare professionals of different roles and professions [22]. The “Situated Learning” theory proposed by Lave and Wenger can contribute to shed light on the relevance of group-learning and competence development within the programme. The theory posits that learning is not merely an individual intellectual process; instead, it is a social process that occurs in collaboration with other individuals who possess different competencies in a community of practice [31]. Moreover, they propose that learning is situated in specific contexts, connected to an activity and occurs through collaboration rooted in sociocultural situations. Furthermore, exploring various perspectives on patient safety, such as individual and system perspectives, can enrich our understanding of group-learning and competence development in clinical settings. According to Aase, a system approach considers health services as interconnected entities where multiple factors contribute to adverse events [23]. By integrating Lave and Wenger’s concepts of ‘situated learning’ and ‘communities of practice’ and Aase’s system perspective on patient safety, we believe that we can gain more profound and valuable insights into the healthcare professionals’ experiences with learning and training in the competence programme and its applicability to clinical practice [23, 31].

## Methods

### Design and study context

We employed a qualitative approach with a descriptive design to explore healthcare professionals’ experiences with learning and training in the programme and its applicability in clinical practice [32]. The study was conducted in a Norwegian municipality featuring rural and densely populated central areas to address real-life challenges in knowledge mobilisation and competence transfer. The competence programme was introduced in the actual municipality in 2020. However, due to the COVID- 19 pandemic, the commencement of the programme and its associated courses was delayed until 2022. In Norway, the healthcare system is organised into two levels of delivery: primary and secondary healthcare services, each governed by distinct funding and regulations [33, 34]. The overall responsibility of the municipality is to ensure the availability of essential health services for all residents within their jurisdiction, which includes nursing homes, home care services, assisted living facilities, personal or practical assistance, and personal support [35]. The primary healthcare workforce includes RNs, assistant nurses, social educators and support staff. RNs are required to have a three-year bachelor’s degree in nursing, while assistant nurses undergo a two-year vocational program, followed by an additional two years of clinical training [36]. Social educators are also required to possess a three-year bachelor’s degree [37]. The role of assistant nurses within the municipal healthcare system has traditionally been associated with limited responsibility, primarily focusing on direct patient care and delegated tasks. In contrast, RNs work within a clearly defined scope of practice that outlines their overall responsibility for patient care [36]. Additionally, social educators in primary healthcare settings have primarily focused on assisting individuals with various functional disabilities, helping them to improve their quality of life and manage their everyday challenges [37]. Approximately 30% of the personnel in primary healthcare services are untrained and lack relevant health-related education. This raises concerns regarding the appropriate allocation of responsibilities and potential gaps in patient care safety [38].

### Recruitment and sample

The study sample comprises 17 healthcare professionals, including 11 RNs, five assistant nurses and one social educator. The study participants were recruited from three primary care services in the municipality: assisted-living facilities, somatic long-term wards and home-care services. They were recruited with assistance from a leading instructor involved in the programme, along with head nurses from these services. The study’s inclusion criteria required that participants had either attended or conducted at least one course in Steps 1–3 of the ClinObsMunicipality programme within the 18 months leading up to this study. Furthermore, participants needed practical experience with the programme’s applicability in clinical practice (see Fig. 1). The exclusion criteria were healthcare workers who were unskilled assistants. We employed a purposive sampling strategy covering professionals varying in age, length of experience, primary care setting and educational background [23]. All study participants were women (Table 1). They were provided with written information about the study’s purpose, and written informed consent was obtained from each individual before data collection.

**Table 1:** Participants – demographic background data

	*n* = 17
**Roles in the Competence Programme**	
Instructor	10
Course Participant	7
**Profession – education**	
Registered Nurse (RN)	11
Assistant Nurse (AN)	5
Social Educator	1
**Departments**	
Somatic longtime wards	10
Assisted living facilities	5
Home care services	2
**Age (average 39 years)**	
20–29	3
30–39	9
40–49	1
50+	4
**Length of experience (average length 13 years)**	
0–5	3
6–10	6
11–20	5
21–30+	3

### Data collection and analysis

The first author (EA) collected data through semi-structured individual interviews with the study participants between January 2023 to September 2023. The interviews were digitally recorded, lasting between 45 and 60 min, and took place at the participants’ workplaces. Guided by a structured interview guide, the interviews covered the experience of learning and training with colleagues and the applicability of the competence-building programme on health professionals’ work and collaboration in clinical practice.

The transcribed interviews constituted the data for this study, which were analysed using Malteruds’ systematic text condensation, a thematic cross-case analysis [32]. The analysis consisted of four steps: (i) overall impression, (ii) identifying and sorting meaning units, (iii) condensation, and (iv) synthesising. In the first step, the authors read the interview transcripts to gain an overall impression of the participants’ experiences with learning and training in the competence-building programme and its applicability to clinical practice; preliminary themes were identified and discussed. In the second step, meaning units from the interviews were identified and sorted into thematic code groups; in the coding, we focused on the participants’ differing experiences while engaging in the programme and the applicability thereof in their clinical practice. In the third step, the meaningful units were condensed. In the fourth step, the condensates were synthesised to develop general descriptions that reflected the main study results. See Table 2. During the analysis, the authors routinely met, discussing preliminary themes and the coding of the data.

**Table 2:** Illustration of the analysis process

Meaning units - preliminary themes	Code groups	Subgroups	Categories
*I think it is beneficial. We complement each other as we learn. You know that one person may have just graduated*,* another has been working for only a few years*,* and others have been in it longer. By sharing different experiences*,* talking with each other*,* and exercising together in a group of three*,* we became more confident in each other. You never felt stupid or anything like that if there were things you didn’t know*. (AN, 53 years old)	Group learning in the competence program	Familiarity with colleagues and instructors	Shared base of competence through practice-based group-learning with colleagues
*It’s more like we’re speaking the same language. Before*,* when I knew NEWS and ABCDE*,* but the team didn’t*,* it was more like giving instructions: ‘Do this and that!’ Now*,* it’s more collaborative*,* instead of me just telling them what to do without them understanding the reasoning behind it.* (RN, 38 years old)	Scoring tools for systemising clinical observations	Standardised language	Enhanced clinical communication: The impact and applicability of standardised language
*It has been helpful for us RNs that the assistant nurses do a bit more independently when we have duty shifts or are on call for the whole house. They don’t just call and say*,* ‘He is unwell!’ Instead*,* you get something more concrete. When we ask*,* ‘Can you take the measurements?’ they don’t panic; they say*,* ‘Yes*,* we can do that!’* (RN, 30 years old)	Collaboration in clinical work across roles and professions	Task shifting between RNs and ANs	From Colleagues to Team– increased Autonomy and Collaboration

## Results

In the analyses, we identified three categories illustrating how healthcare professionals experienced learning and training in the competence-building programme and its applicability to clinical practice: (1) Shared base of competence through practice-based group-learning with colleagues; (2) Enhanced clinical communication: The impact and applicability of standardised language; (3) From colleagues to team: Increased autonomy and collaboration.

### Shared base of competence through practice-based group-learning with colleagues

The participants emphasised that learning and training alongside their colleagues was essential to benefit from the competence programme for primary healthcare services. As colleagues with different educational and professional backgrounds, the group-learning approach provided health professionals access to the same theoretical knowledge and practical skills within observational competencies. This aspect was considered particularly valuable by the participants, allowing them to learn from and with their colleagues and fostering a sense of fellowship applicable to clinical practice. Attending courses with familiar colleagues from their respective units improved their learning process by creating a safe environment for practical exercises and disclosing a lack of competencies, making the training more applicable to clinical practice. Furthermore, observing each other during training provided insight into their colleagues’ skills and weaknesses and influenced their perceptions of one another’s observational competence, reinforcing the collaborative learning experience. One participant stated:

*I think it is beneficial. We complement each other as we learn. You know that one person may have just graduated, another has been working for only a few years, and others have been in it longer. By sharing different experiences, talking with each other, and exercising together in a group of three, we became more confident in each other. You never felt stupid or anything like that if there were things you didn’t know*. (AN, 53 years old)

In this way, engaging in collaborative learning with colleagues was essential for getting the most out of the competence programme for primary healthcare services. Attending courses with familiar colleagues and an instructor who knew them and the context in which they worked facilitated a different and more effective way of developing observational competencies applicable to clinical work compared to participating in courses with strangers. The participants emphasised that familiarity with their instructors allowed personalised feedback and the incorporation of relevant examples and patient cases that resonated with their everyday professional experiences. They noted that this approach allowed them to learn from clinical situations and investigate new strategies for managing them both individually and in collaboration. Participants emphasised that this applicability had a transfer value and affected their patient care in clinical settings. A participant said:

*I feel secure knowing that [the instructor] is conducting this course. We know her very well*,* and she knows us*,* too*,* our weaknesses and strengths. She can tell me that I need to train more*,* unlike someone from the outside who does not know me*. (AN, 62 years old)

Some participants experienced attending the programme with unknown instructors, who often failed to address the practical challenges the participants faced in their clinical work with patients. As a result, the study participants felt that the relevance of the courses was lacking, which led to a perceived decrease in their learning outcomes. Furthermore, some participants who had attended courses facilitated by unknown instructors and health professionals from different units reported a reluctance to speak up and potentially disclose their lack of competencies by asking ‘stupid questions’. Their experiences also shed light on the significance of practice-based group-learning with colleagues for achieving a shared competence base.

### Enhanced clinical communication: the impact and applicability of standardised language

The participants emphasised that introducing standardised scoring tools in the programme to systematise clinical observations and assess patients’ conditions created a common language and terminology applicable to clinical practice, thereby strengthening trust between health professionals within the workplace. After participating in the competence programme, the health professionals’ language was more aligned when describing their clinical observations of patients’ conditions and deterioration. They also experienced a better understanding of each other’s observations. Incorporating tools such as ABCDE, NEWS and ISBAR provided a standardised language and communication framework reference. A participant said:

*It’s more like we’re speaking the same language. Before*,* when I knew NEWS and ABCDE*,* but the team didn’t*,* it was more like giving instructions: ‘Do this and that!’ Now*,* it’s more collaborative*,* instead of me just telling them what to do without them understanding the reasoning behind it.* (RN, 38 years old)

Group-learning and training among healthcare professionals also established a foundation for enhancing clinical self-confidence by promoting a standardised language through scoring tools and communication strategies applicable to clinical practice. According to the participants, this approach enabled a more effective and safer exchange of observations in acute situations, leading to their experience of a notable improvement in managing deteriorating patient situations. They described a newfound sense of control that positively impacted their patient work and safety. One RN explained:

*Earlier*,* I used to get stressed in acute situations when patients were unwell. I could become a bit like a headless chicken*,* flying here and there*. (RN, 61 years old)

Participants noted that using standardised scoring tools and common language improved communication among healthcare professionals across various levels of treatment in cases of the deterioration of a patient’s condition. This approach strengthened the participants’ self-confidence in their interactions with secondary healthcare services and emergency departments when articulating clinical observations and patients’ conditions. Consequently, they experienced a more significant impact during the patient’s treatment as they observed that external professionals took their concerns more seriously. The application of a common language by using scoring tools to observe patients’ conditions systematically was identified as a crucial factor in improving the accuracy and efficiency of communication between healthcare professionals across service levels. RNs reported receiving prompt responses from physicians and nurses in secondary health services, facilitating faster, more effective decision-making in emergencies than relying on subjective descriptions to convey a patient’s condition. RNs from assisted living facilities and somatic long-term wards also indicated that improving internal and external communication increased their ability and efficiency when initiating treatment within a clinical setting. Ultimately, this resulted in enhanced patient care during acute circumstances. In their experience, hospital admissions decreased after implementing a common language using standardised scoring tools and communication strategies in their clinical practice. A participant said:

*We have far fewer admissions than before. Previously*,* we would send them in for something like shortness of breath. Now*,* we give oxygen*,* offer intravenous treatments and treat them. We have done much more since getting these scoring tools. The patients avoid the strain of being transported to [the hospital] only to turn around at the door.* (RN, 61 years old)

Some participants indicated, however, that they needed further training in using scoring tools for effective communication across various service levels. They underscored the necessity of regular group-learning sessions to enhance communication skills and improve care quality for patients in acute conditions.

### From colleagues to team: increased autonomy and collaboration

The participants reported that their involvement in the programme enhanced their autonomy and ability to collaborate in clinical environments. They noted that engaging in group-learning activities was beneficial and provided transferable value to clinical practice as the group-learning activities facilitated a deeper understanding of each other’s roles and responsibilities within the teams, thereby fostering stronger teamwork in clinical settings. Before attending the programme, they expressed uncertainty regarding seeking help from colleagues, as they were unsure of one another’s comfort levels and competencies to perform various tasks. However, participating in group-learning activities allowed them to observe their colleagues’ observational competencies and made it easier to approach the right individuals for support in their everyday work. The participants found this approach relevant to clinical practice as it improved teamwork, facilitated task sharing and made patient care more efficient and seamless.

The participants also emphasised that enhancing competence in clinical observations through group-learning activities led to redistributing responsibilities and tasks between the RNs’ and assistant nurses’ colleagues. This redistribution had both positive and negative notes. On a positive note, assistant nurses could now perform more detailed symptom observations and interpret measurement results independently of the RNs following their collaborative training. This autonomy strengthened their position within the team and led them to take on additional responsibilities in patient care and perform tasks previously assigned to RNs. An assistant nurse from a somatic long-term ward said:

*It feels good to do more of the work myself. You always want to develop*,* even if you’re not highly educated. You have more fun at work when you master it. You feel important in a way. It’s easy just to hand over all the medical tasks to the nurse because you think it’s safest for the patient. That can put much pressure on the RN*,* even though you do it with the best intentions. You think*,* ‘She has much more competence than I have.’* (AN, 24 years old)

According to the RNs, many found it easier to delegate tasks to assistant nurses after learning and training together. The RNs were more confident that the assistant nurses had improved their competencies after observing and gaining a better understanding of their observational competencies. Delegating tasks to assistant nurses reduced the workload for RNs, thereby allowing them to allocate their time and resources more efficiently, such as addressing more complex medical needs. As one RN stated:

*It has been helpful for us RNs that the assistant nurses do a bit more independently when we have duty shifts or are on call for the whole house. They don’t just call and say*,* ‘He is unwell!’ Instead*,* you get something more concrete. When we ask*,* ‘Can you take the measurements?’*,* they don’t panic; they simply say*,* ‘Yes*,* we can do that!’* (RN, 30 years old)

Even though the assistant nurses improved their observational competencies, it was noted on the negative side that they sometimes seemed to have reduced their level of response to critical situations involving deteriorating patients. The RNs attributed this lack of confidence to the assistant nurses’ heightened awareness of the complexities and associated risks. As a result, RNs experienced instances where assistant nurses had returned tasks and responsibilities to them. An RN said:

*The readiness for action may have decreased because nurse assistants now think calling the emergency room is a bit scary. We have been a bit puzzled because we [RNs] aren’t always available*,* so they must manage. That is what we hoped the course could strengthen. But perhaps they have realised how serious it is then. It could be that they see the connection a little more.* (RN, 39 years old)

## Discussion

The results of this study emphasised that health professionals experienced learning and training alongside colleagues in the programme courses to be applicable to their clinical practice. This collaborative approach enabled participants to engage with cases and scoring tools relevant to their clinical settings, improving their observational competencies and communication abilities, which may have positively impacted patient care and safety. Additionally, the findings indicated that healthcare professionals believed engaging in group-learning activities with familiar colleagues enhanced their learning outcomes by cultivating a supportive environment. This collaboration allowed them to learn from and alongside each other and foster a sense of fellowship relevant to clinical practice. Participants noted that these group-activities promoted a deeper understanding of team responsibilities and improved teamwork within clinical settings, ultimately strengthening interprofessional relationships and enhancing patient care outcomes. Furthermore, the participants highlighted that the programme strengthened their clinical confidence and autonomy within primary healthcare settings. Consequently, the redistribution of responsibilities empowered assistant nurses, although the results also illuminated reluctance in their responses to critical situations. Below, we will discuss the impact of these results and the study’s limitations. By integrating Lave and Wenger’s concepts of ‘situated learning’ [31] and Aase’s system perspective on patient safety [23], we can gain deeper insights into healthcare professionals’ experiences with this competence programme and its applicability to clinical practice.

### Previous research: new perspectives

This study is not the first to highlight the importance of group-learning activities within competence-building programmes in primary healthcare settings. Previous research has demonstrated that healthcare professionals in primary healthcare contexts generally view participating in such programmes favourably, as these initiatives enhance their competencies, foster trust and respect among colleagues and improve the quality of patient care [14, 39, 40]. Several studies within hospital contexts show that healthcare professionals find scoring tools like NEWS and ISBAR beneficial [41–43]. These tools improve clinical decision-making, facilitate clear communication between healthcare providers and support early detection of patient deterioration and patient safety. However, studies examining the application of these scoring tools outside hospital settings have identified challenges [44, 45]. It has been highlighted a need for tailored approaches suited to different settings, modifications for specific patient populations, and ensuring consistent use among all healthcare professionals [44, 45]. As with other studies, our findings emphasised the potential benefits of implementing standardised scoring tools to systematise clinical observations in primary care settings. Examples are enhanced communication within teams and with external healthcare parties, increased clinical confidence, and improved management of acute situations.

The complexity associated with competence improvement within primary healthcare settings is well-documented, with previous studies indicating that individual and organisational challenges impact these enhancements [15, 46]. While initiatives aimed at improving observational competencies in nursing homes and home-based care services have shown positive outcomes, the complexity of these programmes can present significant challenges for healthcare professionals [15, 46]. Our study contributes to understanding the significance of collaborative learning activities for strengthening clinical observational competencies applicable to clinical practice and interprofessional collaboration within primary healthcare settings. According to a scoping review and a qualitative study from general practice [4, 47], these factors can significantly improve patient safety.

### Building a shared base of observational competence applicable to clinical practice

Our study showed that learning and training alongside familiar colleagues facilitated a supportive environment for developing and sharing competencies. This setting enabled participants to learn from and with their colleagues, fostering a sense of fellowship applicable to clinical practice. The findings revealed that participants especially valued this aspect, as it provided a more effective means of improving observational competencies than taking courses with strangers. This result aligns with Lave and Wenger’s theory, which posits that learning is a social process occurring through collaboration among individuals possessing different knowledge and skills within a community of practice [31]. Our findings indicated that the collective learning process enhanced colleagues’ trust and promoted teamwork among professionals in primary healthcare settings. This was achieved by improving their understanding of team roles and responsibilities, reducing uncertainties about seeking help and improving collaboration in clinical settings. These findings are consistent with Lave and Wenger’s assertion that developing shared values and competencies within a ‘community of practice’ results in enhanced educational experiences and improved work performance [4]. These dynamics are particularly crucial in primary healthcare, as they facilitate access to shared knowledge and practical skills within observational competencies. This can help reduce variations in care provided and enhance patient safety [23].

Our findings emphasised the importance of competence disclosure among healthcare professionals by revealing a complex interplay between individual knowledge, organisational expectations and patient safety. However, the literature indicates concerns in healthcare settings where the fear of appearing incompetent can impede professional development and compromise patient safety [23]. Research has found that healthcare professionals often encounter difficulty disclosing their lack of competencies, which may stem from a desire to maintain an appearance of competence and avoid negative feedback [48, 49]. Thus, fostering a psychologically safe environment for professionals to express concerns and mistakes without fear of judgment is essential [50]. This aligns with our results, demonstrating that familiar instructors and colleagues emerged as a significant factor when establishing a safe collective learning environment, where vulnerabilities were embraced and competence gaps were respectfully addressed. Drawing on the concepts of situated learning by Lave and Wenger [36], we argue that healthcare professionals may benefit from learning and training with familiar colleagues and instructors, applicable through enhancing collaboration in clinical practice, contributing to what Lave and Wenger call ‘a community of practice’ [31]. By fostering an environment in group-learning settings in which it is acceptable to admit what one does not know, healthcare teams can create a culture of transparency and collective support [51].

The ClinObsMunicipality programme’s introduction of relevant cases, scoring tools and practical tasks allowed healthcare professionals to apply their learning and training directly to patient-care scenarios, thereby enhancing observational competencies with transfer value applicable to clinical practice. This practical application is a core principle of ‘situated learning’, wherein competence is theoretical and practical within the specific context of healthcare delivery [31]. Our findings also illustrate Lave and Wenger’s perspective on ‘situated learning’ [31], demonstrating how healthcare professionals experienced both improving their decision-making competencies in clinical practice through a shared understanding of patient conditions and enhancing their collaboration in clinical practice.

### Collaboration in clinical practice: balancing task delegation and patient safety

The extant literature has identified risks related to patient safety when transferring responsibilities from secondary to primary healthcare settings, showing increased rehospitalisation rates and mortality following these transitions [52–54]. Many Western countries face a shortage of skilled healthcare workers, particularly nurses [17]. This shortage raises concerns about safety and quality in primary healthcare settings and highlights the importance of evaluating the transfer of tasks among health professionals. Exploring various perspectives on patient safety can enrich our understanding of group-learning and competence development in clinical settings. According to Aase, a system approach considers health services as interconnected entities where multiple factors contribute to adverse events [23]. As illustrated in our results, factors contributing to adverse events may include environments where professionals feel reluctant to disclose shortcomings due to fear of negative feedback, which can hinder personal growth and compromise patient safety. Increased awareness of potential consequences may lead to excessive caution, delaying timely interventions. At the same time, a lack of familiarity with scoring tools can lead to misuse or misunderstandings in interprofessional communication. Traditionally, assistant nurses primarily focus on direct patient care and tasks delegated by RNs, while RNs serve as coordinators, providers, and evaluators of care, bearing the overarching responsibility [51, 55]. The findings of our study suggested that group-learning activities increased the observational competencies and autonomy of healthcare professionals in primary healthcare, enabling assistant nurses to assume greater responsibilities and facilitate the redistribution of tasks from RNs. However, the implications of the redistribution of tasks are multifaceted and raise essential patient safety considerations. The results also revealed that assistant nurses hesitated to act in acute situations despite possessing improved observational competencies. This hesitancy may stem from an increased awareness of the potential consequences of their decisions. Although being cautious can positively impact patient safety, it also risks impeding timely interventions in critical circumstances.

According to the system perspective on patient safety, Aase emphasises the importance of interactions among different professional roles and system factors, such as communication tools, when determining patient outcomes in primary healthcare settings [23]. This perspective aligns with our findings, which suggested that a standardised language and terminology using scoring tools can improve communication and, thereby, the accuracy of the information shared among healthcare professionals. By improving the descriptions of clinical observations of deteriorating patients across various professions and service levels, these tools and the standardised language can foster a sense of calm in emergencies. Equipping professionals with the competencies and tools to provide immediate care can reduce the risk of unnecessary hospital transfers and alleviate the burden on patients and healthcare systems [4, 47]. However, the language used in the scoring tools must be clear and understandable for all team members to prevent the risk of a false sense of security; if not, this may contribute to adverse events [44, 45]. Misunderstandings or insufficient familiarity with scoring tools can result in incorrect decision-making and compromise patient safety in primary healthcare settings [23].

### Strengths and limitations

In this study, we explored healthcare professionals’ experiences with a competence programme that focuses on improving healthcare personnel’s observational competencies to enhance patient safety in primary healthcare settings. Our aim has not been to evaluate the participants’ learning outcomes based on the competence programme. Rather, we concentrated on their experiences and perceptions of learning and training alongside their colleagues and its applicability to clinical practice. This approach involved examining their experiences of engaging in courses within the programme and how these interactions may have been translated into practical applications in clinical contexts. By prioritising their subjective insights, we aimed to achieve a deeper understanding of the relevance and impact of the learning and training in the programme on their professional practice and how this may have impacted patient care and safety.

The findings provide valuable insights into delegating tasks from RNs to assistant nurses in primary care, where knowledge is scarce. One limitation of this study could be that it did not include the experiences of workers without healthcare education despite their significant role in primary healthcare. However, only a few assistants were included in the ClinObsMunicipality programme training courses. Some of the assistants who were invited to the study declined, expressing a fear of being evaluated.

The complexity in primary healthcare stems from the diverse needs of patients and the various contexts in which care is delivered. A notable limitation of our study is that the experiences from home-care services came forward on a much smaller scale than those from long-term wards and assisted-living facilities due to recruitment challenges. One reason was that home-care services in this study’s municipality had not advanced in implementing the competence programme. Consequently, home-care study participants had less experience with the programme’s training courses.

We interviewed fewer health professionals who participated in the training programme compared to the number of course instructors. This imbalance may have influenced the study’s findings, as instructors might have different perspectives than the course participants. It is essential to clarify that the instructors did not shift to different departmental roles; they expanded their duties by taking on instructional responsibilities while maintaining their full-time positions as RNs. Additionally, the participants who served as instructors already held advanced positions within their respective departments, with titles such as professional development nurse and coordinating nurse. Thus, it is reasonable to infer that they had a pre-existing interest in professional development and competency enhancement within primary healthcare settings. This background likely enabled them to offer nuanced insights regarding the relevance of the competency programme to clinical practice, underscoring the importance of incorporating their perspectives into the study. However, it is worth noting that the employees who participated in the interviews also elaborated on several topics that the instructors addressed, and we aimed to achieve a balance by incorporating meaningful contributions from assistant nurses, RNs, and RNs who served as instructors in various primary healthcare settings in the study.

The first author of this paper is a nurse with experience in primary healthcare services, but she had no prior experience attending or conducting courses in ClinObsMunicipality. The data collection and analyses were carried out in collaboration with the three co-authors to address potential bias; they were all experienced in qualitative research and had different academic backgrounds in nursing, nursing education and medical sociology.

## Conclusion

The study indicates that healthcare professionals experienced several positive benefits from participating in the competence-building programme, which was applicable to clinical practice. One of the most notable advantages identified by participants was the significant impact of group-learning activities among healthcare professionals in primary care settings. Our results illuminated that this collaborative approach in the courses enabled them to engage with cases relevant to their clinical settings, thereby improving their observational competencies and positively contributing to patient care and safety. Additionally, it enabled participants to learn from one another and cultivate a sense of fellowship relevant to clinical practice. Participants noted that these group activities promoted a deeper understanding of team responsibilities and improved collaboration within clinical settings, ultimately strengthening interprofessional relationships and enhancing patient care outcomes.

Furthermore, the findings suggest that creating a supportive environment through collaborative learning and training with familiar colleagues can strengthen clinical confidence, improve individual autonomy and facilitate the redistribution of responsibilities among RNs and assistant nurses. Fostering a common language by implementing standardised scoring tools to systematise clinical observations, primary healthcare teams can improve interprofessional communication, increase collaboration and ultimately improve patient care. However, the results also revealed a degree of hesitance among assistant nurses in critical situations after attending the programme, illuminating a complex relationship between increased awareness and the urgency of timely interventions. Identifying the specific learning and training needs of healthcare professionals, particularly concerning task delegation and standardised tools, is essential to equip team members to provide quality care within a complex primary healthcare environment.

Insights from this study contribute to the growing body of literature on competence programmes by emphasising the importance of practice-based group-learning among health professionals in primary care. The findings highlight that healthcare professionals experienced that such initiatives enhance observational competencies, standardise communication, and foster teamwork, which may improve patient safety and clinical outcomes in primary healthcare services. There remains a need for further research investigating the perspectives of healthcare professionals trained as instructors. This may generate more knowledge about whether the programme’s competence-building depends on the instructors’ role and structural and interactional barriers and facilitators for implementing the programme.

## Supplementary Information

Supplementary Material 1.

## Data Availability

Data generated and analysed in this study are not publicly available from the corresponding authors due to local data ownership. Aggregated data are available from the corresponding author upon reasonable request.

## Footnotes

1 *Table inspired by KlinObsKommune (ClinObsMunicipality) [22].
