# Supplementary material for: The applicability of a structured learning programme focusing on improving observational competencies to strengthen patient safety: a qualitative study with professionals in primary healthcare services
Source: BMC Health Serv Res. 2025 Apr 15;25:547. doi: 10.1186/s12913-025-12692-y (PMC11998134; doi:10.1186/s12913-025-12692-y)
Supplement: Supplementary file 1 — Supplementary Material 1. [file 12913_2025_12692_MOESM1_ESM.docx]

**Interview guide**

Introduction of the project and the interviewer.

**Personal Information:**

- Gender, age, education, position and workplace?
- How long have you worked in the department?
- When did you participate in the course? Which level?

**Course experience:**

- After finishing the course, I would like to ask you to share your experiences from that day.
- Can you tell me how you felt about attending/carrying out such a course?
- How did you experience learning new skills and knowledge in this way?

**Course Preparation:**

- How did you prepare for the course?
- Can you share some of the learning experiences you got from attending/giving the course?

**Group Work:**

- Who participated with you in the course (their professional background, education)?
- How did your group collaborate during the course?
- What were your experiences of learning new things together?
- Did you find anything difficult, scary, funny or educational?

**Applicability in Daily Clinical Practice:**

- Can you give examples of how you and your colleagues have applied knowledge and/or skills from ClinObsMunicipality in clinical practice?
- How do you collaborate on patient care? Has the collaboration changed in some ways after coursing? If so, please elaborate on this.
- Has your workday changed since the implementation of ClinObsMunicipality? If so, please elaborate on this.
- Can you share examples of applying what you learned from the course in everyday clinical practice?

**Reflections on your role in Daily Clinical practice:**

- Could you share your reflections on your responsibility in your work today?
- Have you taken on/been given any new tasks since the course? If so, please give examples.
- What does it mean to you that some nurses have become instructors and are teaching their colleagues? / What does it mean to you that you have become an instructor and teaching your colleagues?
- Have these new roles among colleagues improved collaboration or created any challenges or differences within your team? If so, please elaborate on this.

**Areas for Improvements:**

- Have you any reflections about how to maintain skills or insights you have acquired from the ClinObsMunicipality
- Any suggestions for changes that you consider essential improvements?

**Final reflections**: Is there anything we haven’t discussed that you think is important and wish to elaborate more on?

Thank you for participating in this interview!
